# ‘Use of antipsychotics in children and adolescents: a picture from the ARITMO population-based European cohort study’

**DOI:** 10.1017/S2045796020000293

**Published:** 2020-04-20

**Authors:** Florentia Kaguelidou, Jakob Holstiege, Tania Schink, Irene Bezemer, Elisabetta Poluzzi, Giampiero Mazzaglia, Lars Pedersen, Miriam Sturkenboom, Gianluca Trifirò

**Affiliations:** 1 Department of Pediatric Pharmacology and Pharmacogenetics, AP-HP, Hôpital Robert Debré, F-75019, Paris, France; 2 INSERM, Clinical Investigations Center, CIC1426, F-75019, Paris, France; 3 University of Paris, Sorbonne Paris Cité, EA 08, Paris, France; 4 Central Institute of Ambulatory Health Care in Germany, Salzufer 8, 10587 Berlin, Germany; 5 Leibniz Institute for Prevention Research and Epidemiology – BIPS, Achterstr. 30, 28359 Bremen, Germany; 6 PHARMO Institute for Drug Outcomes Research, Van Deventerlaan 30-40, 3528, AE Utrecht, The Netherlands; 7 Department of Medical and Surgical Sciences, University of Bologna, Via Irnerio, 48, 40126 Bologna, Italy; 8 Health Search, Italian College of General Practitioners, Florence, Italy; 9 Department of Clinical Medicine – Department of Clinical Epidemiology, Olof Palmes, Allé 43-45, 8200 Aarhus N, Denmark; 10 Julius Global Health, University Medical Center Utrecht, The Netherlands; 11 Department of Biomedical and Dental Sciences and Morphofunctional Imaging, University of Messina, Italy; 12 Department of Medical Informatics, Erasmus Medical Center, Rotterdam, Netherlands

**Keywords:** Antipsychotics, child psychiatry, epidemiology, primary care

## Abstract

**Aims:**

Prevalence of the use of antipsychotics (APs) in the paediatric population is globally increasing. The aim of this study was to describe multinational trends and patterns in AP use in children and adolescents in Europe.

**Methods:**

This was a dynamic retrospective cohort study comprising all children and adolescents (⩽18 years of age). Data were extracted from five population-based electronic healthcare databases in Europe (Denmark, Germany, Italy, the Netherlands and United Kingdom) from 2000 to 2010. Yearly prevalence and incidence of AP use was expressed per 1000 person-years (PYs).

**Results:**

Prevalence increased from 1.44 to 3.41/1000 PYs (2008) in Denmark and from 2.07 to 4.35/1000 PYs in the NL (2009), moderately increased from 2.8 to 3.24/1000 in UK (2009) and from 1.53 to 1.74/1000 PYs in Germany (2008) and remained low from 0.61 to 0.34/1000 PYs in Italy (2010). Similarly, incidence rates increased from 0.69 to 1.52/1000 PYs in Denmark and from 0.86 to 1.49/1000 PYs in the NL, stabilised from 2.29 to 2.37/1000 PYs in the UK and from 0.79 to 0.80/1000 PYs in Germany and remained low from 0.32 to 0.2/1000 PYs in Italy. AP use was highest in 15–18 year olds and in boys compared to girls. Yet, the use observed in the 5–9 year olds was found to be comparatively high in the NL. Prescriptions of second generation APs, especially risperidone, were privileged but the first generation APs were still prescribed in the youngest.

**Conclusions:**

A steady increase in AP use in children and adolescents was observed essentially in the NL and Denmark. The use in Germany and Italy was lowest among countries. The use of APs under 9 years of age underlines their off-label use and should be carefully monitored as the risk/benefit ratio of these medications remains unclear in young children. AP use was altogether lower in Europe as compared to that reported in North America.

## Introduction

Antipsychotic (AP) medications are effective in treating several psychiatric conditions in children and adolescents. Although not curative, they allow adequate control of clinical symptoms in lifelong psychiatric diseases (Caccia *et al*., 2011). Yet, the use of APs is associated with a substantial number of adverse effects in this population and different agents present highly variable safety profiles (Pringsheim *et al*., 2011; Seida *et al*., 2012). Therefore, prescribing of APs involves a difficult balance between the need to relieve mental disease symptoms and the risk of drug-induced toxicity.

Over the past three decades, studies have consistently demonstrated that the prevalence of the use of APs and duration of the AP therapy is increasing over time in the pediatric population (Vitiello *et al*., 2009; Steinhausen and Bisgaard, 2014; Halfdanarson *et al*., 2017). Despite marketing authorisations for the use of some first-and second-generation antipsychotics (FGAs and SGAs) specifically in children and adolescents (Kaguelidou and Acquaviva, 2016), the vast majority of these medications are still prescribed ‘off-label’ in the pediatric population. The observed increase in the use of APs probably reflects the ‘off-label’ prescribing of APs in non-psychotic disorders, such as attention deficit hyperactivity disorder (ADHD) or disruptive behaviour, and in children younger than the approved age ranges (Penfold *et al*., 2013).

However, the majority of these drug utilisation studies are based on data from Northern American countries and information on population-based use of these medications in Europe is more limited (Zoega *et al*., 2009; Verdoux *et al*., 2010; Penfold *et al*., 2013; Steinhausen and Bisgaard, 2014; Verdoux *et al*., 2015; Waszak *et al*., 2018). Differences in the diagnosis and management of psychiatric conditions as well as in the attitude to prescribe APs may hinder extrapolation of results from one continent to the other. In fact, with regard to the use of SGAs, a wide inter-country variability was observed in a study from the year 2000, where dispensations of SGAs represented 66% of total AP use in children and adolescents in the US *v*. 48% in the Netherlands and only 5% in Germany (Zito *et al*., 2008).

Therefore, the aim of this study was to describe the prevalence and incidence of AP use in children and adolescents in five European countries.

## Methods

### Data sources

Data were extracted from five population-based electronic healthcare databases in Europe. The Health Improvement Network (THIN) is a database of primary care medical records of about 5.9 million patients from 500 general practices (GPs) in the United Kingdom (UK). The PHARMO Database Network is a patient-centric data tracking system that captures medical information, including information on drug dispensing, for approximately 4 million inhabitants in 65 municipal areas in the Netherlands (NL). The Aarhus University Hospital Database comprises medical information including hospital and outpatient visits from 1.8 million inhabitants in Denmark (DN). The German Pharmacoepidemiological Research Database (GePaRD) consists of claims data from four German statutory health insurance providers, three of which accepted to contribute data for this study resulting in a source population of 8 million insurants. Finally, the Emilia Romagna Regional database (ERD) is a claims database that contains information on all reimbursable healthcare services, including drugs, for about 4.5 million inhabitants of the Emilia Romagna region in Northern Italy (IT). These databases contain information from the healthcare records of almost 27 million European citizens. THIN contain records from GP, while PHARMO, AARHUS, GePARD and ERD are comprehensive administrative/record-linkage systems in which drug dispensing data for a well-defined population are linked to a registry of hospital discharge diagnoses and various other registries.

All databases and their content have been extensively described in previous ARITMO publications and have already been used for the conduct of pharmacoepidemiological studies in compliance with European guidelines for the use of medical data for research (Holstiege *et al*., 2015; Mor *et al*., 2015; Oteri *et al*., 2016). Also, methodological aspects of multiple database studies carried out in ARITMO and other EU funded projects have been fully described in a previous publication (Trifiro *et al*., 2014). Data were analysed using a distributed network approach, in which data holders maintain control over their original data and only anonymised and aggregated data are shared. This was done through the preparation of data according to a common data input model followed by local data aggregation using custom-built software, Jerboa^©^ (Trifiro *et al*., 2014). The respective scientific and ethics committees of each database approved the conduct of the study. With regard to GePaRD, the use of data was approved by the statutory health insurance providers and their authorities.

### Study design and population

This was a dynamic retrospective cohort study. The study population comprised all children and adolescents (⩽18 years of age), registered with the databases during the study period with at least 1 year of valid data (except for newborns). The period for data collection differed between databases. It was longer in NL and UK (2000–2009) followed by DN (2001–2008), Germany (2005–2008) and IT (2006–2010). Children were followed from the start of the study period or, if later, the start of entry into the database until their 19^th^ birthday, the end of the study period, exit from the database, death or latest data recorded, whichever came first.

### Antipsychotic medications

All drugs under the ‘N05A’ pharmacological subgroup of the Anatomic Therapeutic Chemical classification system (except for lithium [N05AN]) were included. APs were sub-classified into second-generation (clozapine, olanzapine, quetiapine, asenapine, sulpiride, amisulpride, risperidone, aripiprazole, paliperidone, iloperidone, ziprasidone and sertindole) and first-generation agents (all the remaining). AP exposure was assessed using dispensing and/or prescription data from all databases.

### Statistical analysis

Prevalence of AP use was defined as the number of children and adolescents that received at least one AP drug dispensing divided by the number of person-years (PYs) of follow-up in the study period and expressed as rate per 1000 PYs. Incidence of AP use was defined as the number of ‘new antipsychotic users’ per 1000 PYs. ‘New users’ were children and adolescents that had a first prescribing or dispensing of any AP drug after a drug-naïve period of 1 year. Because of the dynamic nature of the population we used PYs rather than the total number of individuals as denominators. Of note, PYs of exposure of prevalent users were not included in the denominator for calculation of the incidence of use. Both prevalence and incidence of use were estimated per calendar year and stratified by sex, age group (⩽4, 5–9, 10–14 and 15–17 years) and country. Prevalence and incidence estimates are provided with 95% confidence intervals (95% CI) calculated according to the asymptotic method based on a normal approximation. Relative changes (RC) in prevalence and incidence rates over the study period were expressed as percentage changes and calculated for each country as the difference in prevalence or incidence between the respective first and last year for which data was available, divided by the prevalence or incidence in the first data year.

Dispensing frequency by class of AP agents (FGAs and SGAs), age group and country was also measured. The number and type of APs covering 90% of all AP dispensing (DU 90%) were estimated by age group and country.

Data were described in narrative and tabular forms. Statistical analyses were performed using the SAS software version 9.3 software (SAS Inc, Cary, North Carolina).

## Results

During the study period, average annual study population and population exposed to APs were respectively: 336.576 and 752 children and adolescents in DN, 1.340.163 and 1.949 in Germany (GE), 768.631 and 2.206 in UK, 798.431 and 2.149 in NL and 760.866 and 342 in IT.

**Fig. 1.**
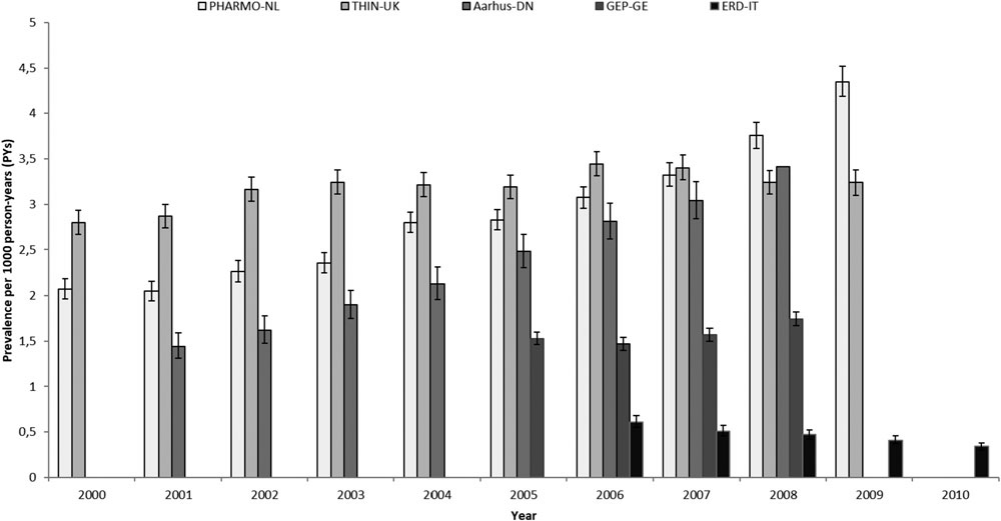
Prevalence of AP use by country and calendar year. PHARMO-NL: *PHARMO* Database Network, the Netherlands. THIN-UK: *The Health Improvement Network*, United Kingdom. Aarhus-DN: *Aarhus University Hospital Database*, Denmark. GEP-GE: *German Pharmacoepidemiological Research Database* (GePaRD), Germany. ERD-IT: *Emilia Romagna Regional database*, Italy.

**Fig. 2.**
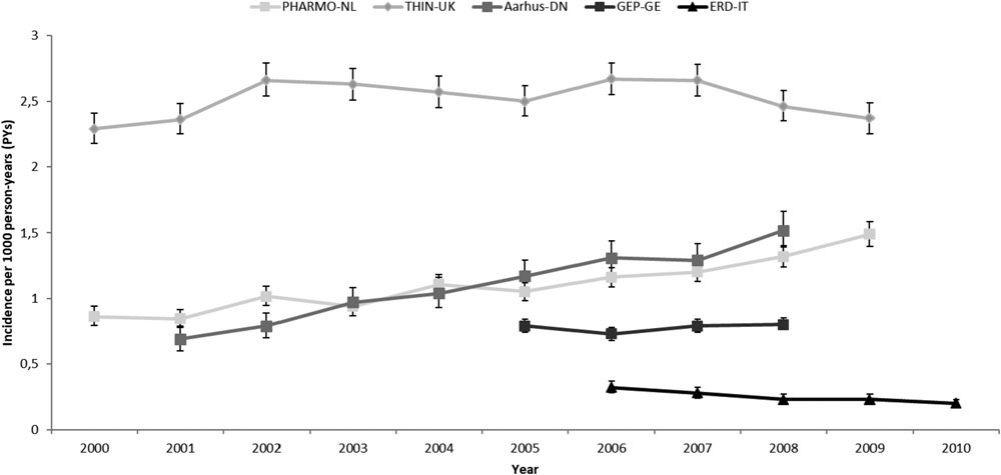
Incidence of AP use by country and calendar year. PHARMO-NL: *PHARMO* Database Network, the Netherlands. THIN-UK: *The Health Improvement Network*, United Kingdom. Aarhus-DN: *Aarhus University Hospital Database*, Denmark. GEP-GE: *German Pharmacoepidemiological Research Database* (GePaRD), Germany. ERD-IT: *Emilia Romagna Regional database*, Italy.

Prevalence and incidence of AP use per country and calendar year with 95% CI are shown in Figs 1 and 2, respectively.

Increases over time in prevalence and incidence rates were observed in DN and NL. In DN, prevalence increased from 1.44 (CI 95%: 1.31–1.58; 2001) to 3.41/1000 PYs (CI 95%: 3.21–3.62; 2008) (+137% RC) and in NL, from 2.07 (CI 95%: 1.96–2.18; 2000) to 4.35/1000 PY (CI 95%: 4.19–4.52; 2009) (+110% RC). Incidence rates increased from 0.69 (CI 95%: 0.6–0.79; 2001) to 1.52/1000 PY (CI 95%: 1.39–1.66; 2008) (+137% RC) in DN and from 0.86 (0.79–1.93; 2000) to 1.49/1000 PYs (1.39–1.58; 2009) in NL (+73% RC).

In UK and Germany, no increase in the use of APs was seen. In UK, prevalence and incidence changed respectively, from 2.8 (CI 95%: 2.67–2.93; 2000) to 3.24/1000 PYs (CI 95%: 3.1–3.38; 2009) (+16% RC) and from 2.29 (CI 95%: 2.18–2.41; 2000) to 2.37/1000 PYs (CI 95%: 2.25–2.49; 2009) (+3.5% RC). Of note, the UK had the highest incidence rates observed among all countries. In Germany, prevalence changed from 1.53 (CI 95%: 1.46–1.6; 2005) to 1.74/1000 PYs (CI 95%: 1.67–1.82; 2008) (+14% RC) and incidence remained stable from 0.79 (CI 95%: 0.74–0.84; 2005) to 0.8/1000 PYs (CI 95%: 0.75–0.85; 2008) (+1.2% RC).

The only country where both prevalence and incidence of AP use slightly decreased over the years was IT. Prevalence amounted to 0.61 (CI 95%: 0.55–0.68) in 2006 and 0.34/1000 PYs (CI 95%: 0.3–0.38) in 2010 (−44% RC) and incidence was 0.32 (CI 95%: 0.28–0.37) in 2006 and 0.2/1000 PYs (CI 95%: 0.17–0.23) in 2010 (−37.5% RC).

**Fig. 3.**
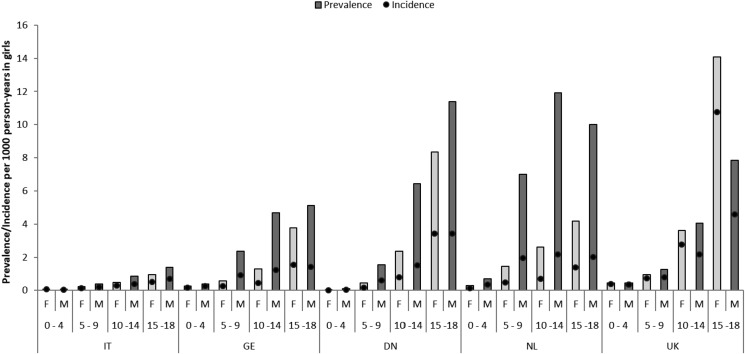
Prevalence and incidence rates per sex, age group and country. NL: the Netherlands; UK: United Kingdom; DN: Denmark; GE: Germany; IT: Italy; F: female users; M: male users.

During the entire study period, the overall use (both prevalence and incidence) of APs was higher with increasing age, with a maximal use observed between 15 and 18 years of age, and more prevalent in boys than girls at all ages (Fig. 3). However, maximal prevalence and incidence of use was observed in boys between 10 and 14 years of age in NL and in girls between 15 and 18 years in the UK. Moreover, the use of APs in children ⩽4 years of age was null in Denmark, limited in IT and the highest in NL followed by UK and Germany.

**Fig. 4.**
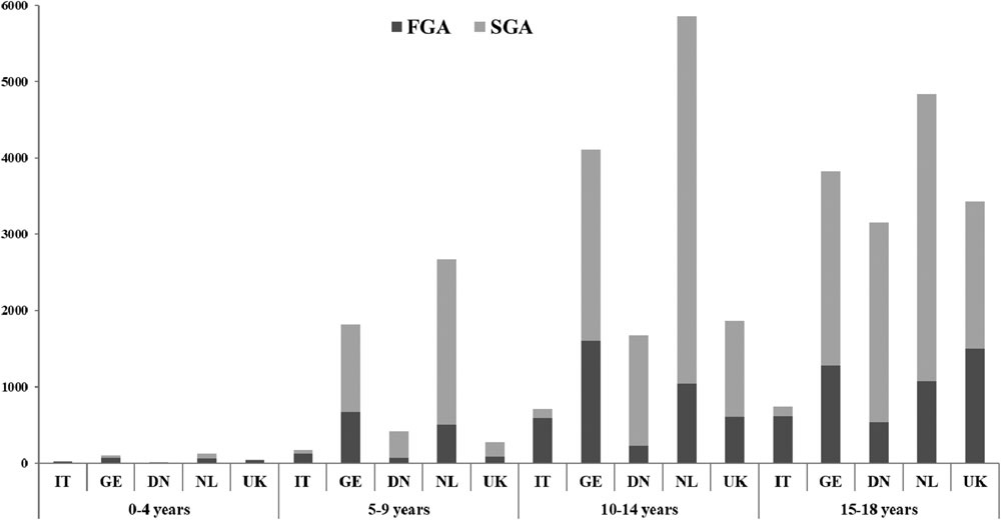
Frequency of dispensing of AP drugs by type of AP, age group and country for year 2008. FGA: first generation antipsychotics, SGA: second generation antipsychotics. NL: the Netherlands; UK: United Kingdom; DN: Denmark; GE: Germany; IT: Italy

The frequency of AP dispensing by type of AP, age group and country for the year 2008 are illustrated in Fig. 4. Overall, SGAs were more frequently prescribed than FGAs and this is most obvious in the latest data years in all countries (Table 1). Yet, wide variation across different age groups was noted. In the youngest (⩽4 years), FGAs were clearly preferred with the exception of DN (22% of all prescriptions in 2008). The use of FGA decreased with age and years of study accounting for less than 50% of prescriptions in children above 4 years of age in all countries except IT in the most recent data-available year. In IT, FGAs remain frequently prescribed, 89% of all prescriptions in 5–9 years, 86% in 10–14 years and 78% in the 15–18 years age group. Of note, in the UK, FGA still accounted for 44% of all AP prescriptions in adolescents between 15 and 18 years of age, a percentage much higher than those observed in the other countries with the exception of IT. Variability in the use of FGA and SGA between countries and age groups is shown in Fig. 4 for the calendar year 2008 which was the only year with data available from all databases.

**Table 1.** Percentage of FGA prescriptions on total AP prescriptions in the first and the last available data years in the different countries

		First available data year	Last available data year	
		Percentages (%)	Percentages (%)	
0–4 years	IT	84	100	IT: 2006–2010
	GE	93	66	GE: 2005–2008
	DN	100	22	DN: 2001–2008
	NL	83	63	NL: 2000–2009
	UK	99	100	UK: 2000–2009
5–9 years	IT	77	89	
	GE	63	37	
	DN	83	15	
	NL	72	15	
	UK	79	17	
10–14 years	IT	89	86	
	GE	53	39	
	DN	66	17	
	NL	66	15	
	UK	78	28	
15–18 years	IT	81	78	
	GE	36	33	
	DN	48	17	
	NL	54	21	
	UK	71	44	

Ninety percent of all AP prescriptions (DU90%) in the population were covered by 9 drugs in IT, 8 in Germany, 6 in DN, 4 in NL and 5 in UK. Although, the number of drugs accounting for the DU90% increased with age, it is noteworthy that in the 0–4 years age group the number of prescribed drugs was higher than that in the 5–9 years group (Table 2). Risperidone is the most frequently prescribed AP in all countries with the exception of IT where chlorpromazine is generally prescribed at all ages. Also, prochlorperazine, an AP with mainly antiemetic properties, was the most commonly prescribed drug in the UK at all ages also prescribed in the NL in the ⩽4 years age group.

**Table 2.** APs that cover 90% of all prescriptions per country and age group during available data years

IT		Germany		DN		NL		UK	
Drug	%	Drug	%	Drug	%	Drug	%	Drug	%
0–4 years									
Chlopromazine	32.7	Pipamperone	28.3	Levomepromazine	39.1	Moperone	59.0	Prochlorperazine	85.3
Haloperidol	12.2	Promazine	28.1	Chlorprothixene	32.3	Risperidone	22.3	Risperidone	4.6
Pimozide	10.2	Risperidone	16.4	Risperidone	28.6	Haloperidol	8.2	Haloperidol	4.3
Levomepromazine	9.3	Levomepromazine	6.6			Prochlorperazine	2.5		
Periciazine	9.3	Melperone	5.6						
Clotiapine	8.3	Chlorprothixene	3.8						
Amisulpride	5.4	Tiapride	3.6						
Quetiapine	3.4								
5–9 years									
Chlopromazine	25.7	Risperidone	52.5	Risperidone	68.3	Risperidone	64.2	Risperidone	48.6
Haloperidol	18.0	Pipamperone	22.7	Pimozide	17.4	Pipamperone	26.3	Prochlorperazine	39.4
Periciazine	16.0	Tiapride	15.5	Chlorprothixene	8.5			Thioridazine	4.0
Levomepromazine	8.3								
Risperidone	6.8								
Olanzapine	6.6								
Pimozide	5.1								
Quetiapine	3.8								
10–14 years									
Chlopromazine	22.0	Risperidone	47.0	Risperidone	59.9	Risperidone	59.2	Risperidone	50.4
Haloperidol	14.1	Tiapride	21.3	Pimozide	15.2	Pipamperone	23.7	Prochlorperazine	32.8
Pimozide	13.5	Pipamperone	18.0	Chlorprothixene	10.4	Pimozide	5.0	Haloperidol	4.9
Periciazine	13.3	Olanzapine	1.8	Quetiapine	3.6	Olanzapine	3.0	Thioridazine	2.0
Levomepromazine	10.9	Chlorprothixene	1.8	Ziprasidone	3.6				
Risperidone	10.1	Quetiapine	1.8						
Clotiapine	6.9								
15–18 years									
Chlopromazine	22.6	Risperidone	32.0	Risperidone	39.9	Risperidone	46.3	Prochlorperazine	40.2
Haloperidol	14.1	Pipamperone	11.0	Chlorprothixene	14.3	Pipamperone	18.8	Risperidone	29.1
Clotiapine	14.0	Quetiapine	10.6	Olanzapine	13.9	Olanzapine	10.0	Olanzapine	9.8
Periciazine	9.9	Olanzapine	9.2	Quetiapine	9.2	Quetiapine	7.7	Quetiapine	4.3
Levomepromazine	8.2	Tiapride	7.5	Pimozide	4.4	Pimozide	5.2	Chlopromazine	3.4
Pimozide	7.6	Chlorprothixene	4.1	Aripiprazole	4.1	Haloperidol	4.7	Haloperidol	2.8
Risperidone	6.3	Clozapine	4.0	Levomepromazine	3.9			Sulpiride	1.8
Olanzapine	3.9	Melperone	3.7	Ziprasidone	3.6				
Quetiapine	2.7	Aripiprazole	2.7						
Amisulpiride	2.3	Levomepromazine	2.5						
		Sulpiride	2.4						
		Perazine	2.3						

## Discussion

This study provides a comprehensive overview of the use of APs in children and adolescents in five European countries: Denmark, Germany, Italy, the Netherlands and the United Kingdom. Prevalence and incidence of use varied widely between countries. The use was globally more important among adolescents however, that observed in younger age groups (5–9 years) was found to be comparatively high in the Netherlands. Prescriptions of SGAs were privileged over the years except for the youngest age groups where clinicians still favoured FGAs with the exception of one country.

In the recent years, several studies have alerted on the increasing rate of AP use worldwide in children and adolescents (Halfdanarson *et al*., 2017; Kalverdijk *et al*., 2017) yet, there are few published AP utilisation studies in Europe. The present study showed that the point prevalence of AP use almost doubled over the years in countries like the NL and DN; however, the increase was moderate in the UK and Germany and the prevalence rate actually decreased in IT. These results are in accordance with some previously published studies. In the NL, estimations of prevalence in a previous study ranged from 3‰ in 1997 to 6.8‰ in 2005 (Kalverdijk *et al*., 2008). Accordingly, in DN where the prevalence of use was very low in 1996 (0.3‰), it increased over 6-fold in 2010 (Steinhausen and Bisgaard, 2014). For these two countries, increasing rates were also reflected in the incidence rates and were confirmed in more recent international utilisation studies that covered larger study periods and different databases (Halfdanarson *et al*., 2017; Kalverdijk *et al*., 2017). It is highly probable that this is related to frequent ‘off-label’ prescription of APs, particularly in the youngest, mainly to treat ADHD, conduct and behavioural disturbances (aggression, self-injury, disruptive behaviour, etc.) and mood disorders without solid efficacy and safety evidence for this practice (Penfold *et al*., 2013; Baribeau and Anagnostou, 2014; Hawton *et al*., 2015; Loy *et al*., 2017). Nevertheless, increasing public awareness of pedopsychiatric disorders, greater acceptance of the use of psychotropic medications in children and a subsequently increasing demand for quickly effective therapies in these countries may stimulate AP prescribing. National pedopsychiatry therapeutic guidelines can also explain such practices and inter-country differences though most of the guidelines in Europe plead for non-pharmacological therapeutic approaches especially in AP ‘off-label’ indications such as ADHD or neurotic disorders (Taylor *et al*., 2004; Hodgkins *et al*., 2013). Regulatory approvals for APs are also harmonised across Europe and thus less susceptible to explain inter-country differences in use. Therefore, reasons for increasing AP use in NL and DN should be specifically explored.

In the UK, prevalence of AP use doubled between 1992 and 2005 (0.39 to 0.77‰) (Rani *et al*., 2008) but our estimations, although comparatively higher, showed that the increase between 2000 and 2009 was slight. Apart from the differences in study period and data sources used, another possible explanation for the higher prevalence estimation in the present study is that it also included the use of prochlorperazine, which is an AP frequently prescribed in the UK as an antiemetic agent. The latter may also explain why the number of new AP users was close to the number of prevalent users in the country and that incidence of use was the highest observed among all countries. In Germany, recent studies described prevalence ranging from 2.03‰ in 2006 to 2.61‰ in 2011 (Schroder *et al*., 2017) and 3.3‰ in 2012 (Kalverdijk *et al*., 2017). Our prevalence estimations are lower which is probably related to the difference in study periods, study populations and/or methodological approaches. Nonetheless, they described the same discreet increment in use over the years. Both in the UK and in Germany, incidence rates were unchanged during the study period. Hence, the slight increases in prevalence observed were most probably due to an increase in the duration of the AP treatment rather than an increase in the number of prescriptions to newly diagnosed users.

Finally, a previous Italian study described a decrease in the prevalence of AP use from 0.63‰ in 1998 to 0.53‰ in 2004 (Clavenna *et al*., 2007) and this tendency was confirmed in our updated data. Globally, the increase in the use of APs observed worldwide has been largely attributed to their off-label use in pedopsychiatry. However, it is possible that in Italy other classes of psychotropic drugs such as antidepressants may be more largely prescribed especially in behaviour disorders or autism (Clavenna *et al*., 2007; Clavenna *et al*., 2011; Piovani *et al*., 2016). Also, the low prevalence of AP use and the very low prescription rate of SGAs observed in the ERD might be explained by the fact that, in Italy, these drugs are partially dispensed through direct distribution from local psychiatric services and cannot be captured in the outpatient pharmaceutical dispensing flow. As a consequence, an underestimation of the use of APs especially SGA may have occurred.

Altogether, European prevalence rates were lower than those described in the US (39.4‰ per 2-year interval) (Cooper *et al*., 2006) but in Canada, prevalence is comparable to that in the NL (1.66 in 1996 to 6.37% in 2001) (Ronsley *et al*., 2013) although data were not available on the same study period.

Another interesting finding was the age distribution of AP prescriptions. Countries like NL have a very high use of APs in the youngest age groups (0–4 years and 5–9 years) followed by the UK, Germany and Denmark whereas in IT, use is very low in children of less than 9 years of age. Onset of psychotic disorders typically occurs in adolescence as opposed to conduct or autistic disorders associated with aggressive behaviour that can be detected in rather young ages. Therefore, high use in the youngest underlines the off-label use of AP agents for the treatment of a wide range of psychiatric illnesses without solid efficacy evidence and despite major concerns regarding their safety profile especially in very young children (Fraguas *et al*., 2011; Ho *et al*., 2011; Kaguelidou and Acquaviva, 2016; Pisano *et al*., 2016). The extent of the off-label use highly varies between countries and it would be useful to monitor this use by assessing the ratio of prevalence rates as described in this study. On the other hand, gender differences with a higher use in boys compared to girls have been observed as expected in these pathologies (Ronsley *et al*., 2013; Kalverdijk *et al*., 2017). The only exception was in the UK where girls in the 15–18 years of age had the higher prevalence and incidence of use mainly due to the prescription of prochlorperazine.

The nature and the number of prescribed AP agents also varied among countries and age groups. Altogether, there is a clear tendency in limiting FGAs prescribing over the years. Risperidone was the most frequently prescribed SGA in almost all countries. This is highly consistent with numerous national and international AP utilisation reports (Rani *et al*., 2008; Ronsley *et al*., 2013; Halfdanarson *et al*., 2017). Switch in the early 2000s was initially triggered by the fact that SGAs were marketed as being overall safer than FGAs. Since then, SGAs have indeed been associated with a lower risk of neurological adverse reactions than FGAs but they have been clearly associated with a higher risk of weight gain and metabolic abnormalities in both adults and children (Maher *et al*., 2011; Seida *et al*., 2012; Caccia, 2013). Also, despite initial expectations, variability in APs' safety profile appears to be greater among specific SGA agents than between FGA and SGA classes (Fraguas *et al*., 2011; Masi and Liboni, 2011; Kaguelidou and Acquaviva, 2016). Yet, based on our findings, the switch from FGAs to SGAs concerned essentially the older paediatric age groups. In the youngest groups, FGAs were still commonly prescribed even in the most recent study years underlining the fact that in paediatric medical practice, new molecules without specific marketing authorisation are initially prescribed ‘off-label’ in adolescents and older children until more knowledge becomes available on their efficacy and safety. Certainly, inter-country differences observed in the choice of prescribed molecules are directly related to differences in market availability, variation in the diagnosis of paediatric psychiatric disorders, prescribers' habits and therapeutic approaches.

The results of the present study should be considered in view of some limitations. Firstly, we used outpatient prescription/dispensing data and had no information about the actual adherence of patients to their treatment and therefore, the real use of these medications. In addition, use in inpatient/institutionalised children and adolescents has not been assessed although it is unlikely that an AP therapy initiated in an hospital/institution would be discontinued in the outpatient setting especially since recommendations urge to minimise inpatient journeys in minors. Secondly, we do not directly compare national utilisation data given the differences in the nature of corresponding databases. Also, we acknowledge quantitative differences with some more recent studies published in the field. However, despite such differences, conclusions remain the same; some European countries have an already high and increasing rate of AP use (north of Europe) where in others, including UK and Germany, the rates are less important and increasing in a slower pace. In addition, we present data from Italy, a country that had not been previously included in multinational studies. Finally, we did not describe AP polypharmacy or therapeutic associations with other psychotropic drug classes as this was beyond the scope of the present study.

## Conclusions

The use of APs varies widely among European countries, with some presenting an increase in use over the years and others, a stabilisation or slight decrease. While the use is overall higher in adolescents, in some countries there is a clear increase of AP prescribing in younger children. SGAs overall dominate prescribing preferences but FGAs are still prescribed in the youngest in varying proportions. The use of APs in children of ⩽9 years of age underlines their off-label use and should be carefully monitored as the risk/benefit ratio of these medications remains unclear especially in the youngest. Future studies should focus on exploring factors that drive AP use in children and adolescents in each country specifically. As the regulatory context regarding APs is quite homogenous in Europe, it is possible that societal and parental awareness and demands as well as prescribers' preferences play a determining role.
